# Frailty assessment in emergency medicine using the Clinical Frailty Scale: a scoping review

**DOI:** 10.1007/s11739-022-03042-5

**Published:** 2022-07-21

**Authors:** Christophe Alain Fehlmann, Christian Hans Nickel, Emily Cino, Zinnia Al-Najjar, Nigèle Langlois, Debra Eagles

**Affiliations:** 1 Division of Emergency Medicine, Department of Acute Medicine, Geneva University Hospitals, Geneva, Switzerland; 2 School of Epidemiology and Public Health, University of Ottawa, Ottawa, ON Canada; 3 Ottawa Hospital Research Institute, Ottawa, ON Canada; 4 Emergency Department, University Hospital Basel, University of Basel, Basel, Switzerland; 5 Faculty of Medicine, University of Ottawa, Ottawa, ON Canada; 6 Health Sciences Library, University of Ottawa, Ottawa, Canada; 7 Department of Emergency Medicine, University of Ottawa, Ottawa, ON Canada

**Keywords:** Frailty, Clinical Frailty Scale, Older patients, Geriatric, Emergency medicine

## Abstract

**Background:**

Frailty is a common condition present in older Emergency Department (ED) patients that is associated with poor health outcomes. The Clinical Frailty Scale (CFS) is a tool that measures frailty on a scale from 1 (very fit) to 9 (terminally ill). The goal of this scoping review was to describe current use of the CFS in emergency medicine and to identify gaps in research.

**Methods:**

We performed a systemic literature search to identify original research that used the CFS in emergency medicine. Several databases were searched from January 2005 to July 2021. Two independent reviewers completed screening, full text review and data abstraction, with a focus on study characteristics, CFS assessment (evaluators, timing and purpose), study outcomes and statistical methods.

**Results:**

A total of 4818 unique citations were identified; 34 studies were included in the final analysis. Among them, 76% were published after 2018, mainly in Europe or North America (79%). Only two assessed CFS in the pre-hospital setting. The nine-point scale was used in 74% of the studies, and patient consent was required in 69% of them. The main reason to use CFS was as a main exposure (44%), a potential predictor (15%) or an outcome (15%). The most frequently studied outcomes were mortality and hospital admission.

**Conclusion:**

The use of CFS in emergency medicine research is drastically increasing. However, the reporting is not optimal and should be more standardized. Studies evaluating the impact of frailty assessment in the ED are needed.

**Registration:**

https://doi.org/10.17605/OSF.IO/W2F8N

## Introduction

Frailty is a physiological state where small perturbations in health result in disproportionate adverse effects due to an underlying decline in reserve of multiple physiological systems [1–3]. It is common in older Emergency Department (ED) patients with reported prevalence rates between 21 and 62 [4–7]. Frailty is associated with a wide range of adverse outcomes, including mortality [8], hospitalization [9], delirium [7] and diminished quality of life [10]. People often present to the ED due a change in health status, this offers a unique opportunity to alter their health trajectory. To meet the needs of the growing population of older adults with frailty presenting to the ED, there is advocacy for the integration of ED frailty evaluation [11, 12]. However, the benefit and harms associated with frailty screening in the ED are largely unknown [13, 14]. Furthermore, frailty identification in the ED is not common [15]. Cited barriers included feasibility of tools in the time pressured ED environment, lack of formal clinical frailty guidelines for the ED and geriatric expertise [11, 13, 15, 16].

Previous scoping reviews on frailty in the acute care setting have included multiple medical disciplines including geriatrics, emergency medicine, general medicine, cardiology and orthopedics [14, 17]. Van Dam et al. recently completed a narrative review of frailty assessment in the ED [18]. They focused on the predictive accuracy of frailty screening tools, the use of clinical gestalt to determine frailty, and the rationale for and implementation of frailty assessment in the ED. However, some of included studies have used tools that were initially designed to predict risk of adverse outcome (ie ISAR, TRST) and not frailty specifically [5, 19].

**Fig. 1:**
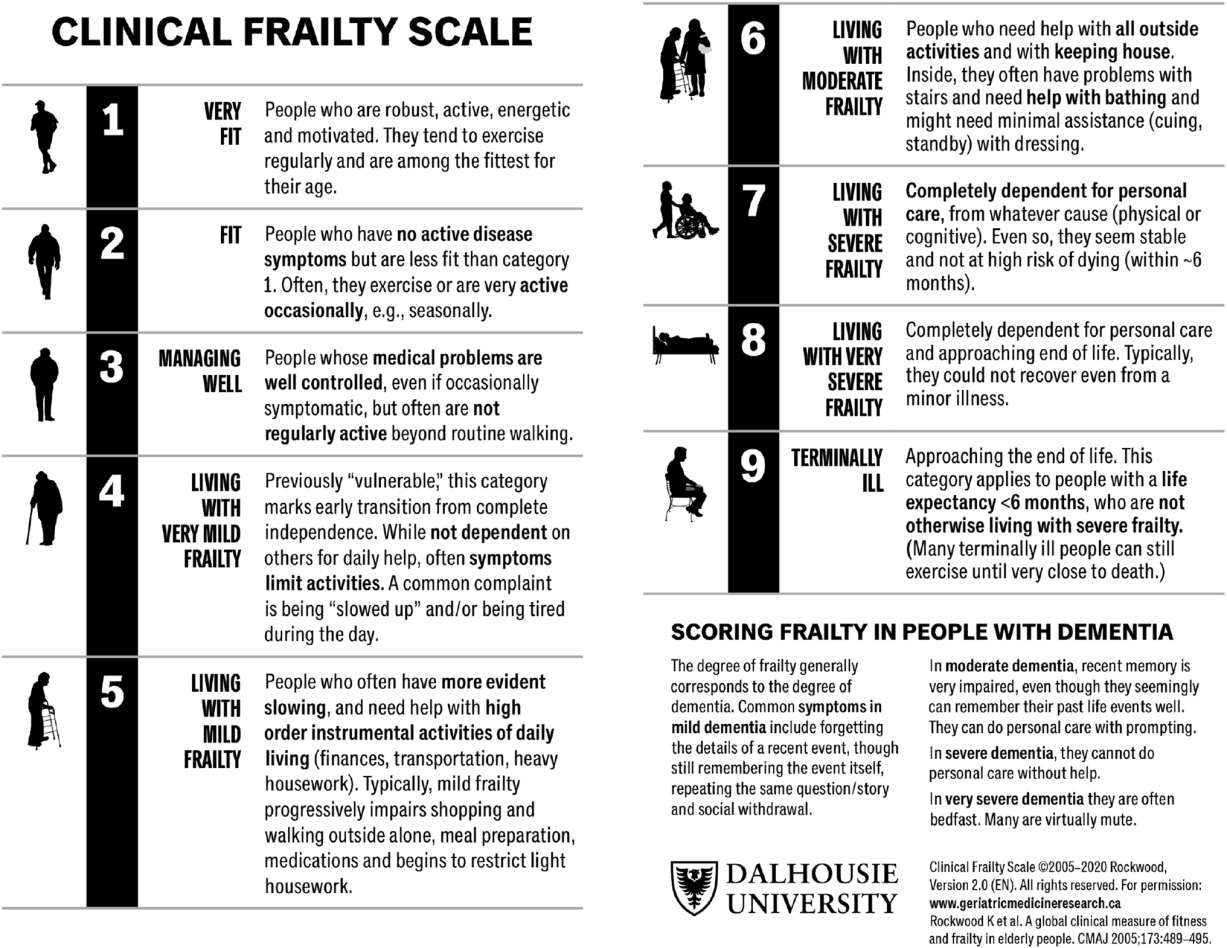
The Clinical Frailty Scale

There are 89 different measures that have been used to evaluate frailty in the acute care literature [20]. The Clinical Frailty Scale (CFS) is one of the most commonly used tools. The CFS was initially a seven-point scale used as a judgment-based tool to assess frailty [21]. In 2007, it was expanded to a nine-point scale, from 1 (very fit) to 9 (terminally ill) (Fig. 1). Compared to other frailty tools, the CFS seems to be the ideal choice for measuring frailty in emergency medicine, because it is easier and faster to use, without giving up any prognostic accuracy [22]. There are no studies that exclusively synthesize information on the use of CFS in emergency medicine. This scoping review is intended to fill this gap, by focusing strictly on the CFS literature in the emergency medicine setting. We aimed to describe the current evidence and identify gaps in knowledge including: version of CFS, timing of CFS evaluation, who is completing the evaluation, goals of frailty evaluation, the prevalence of frailty, and the outcomes associated with frailty identification using the CFS.

**Fig. 2:**
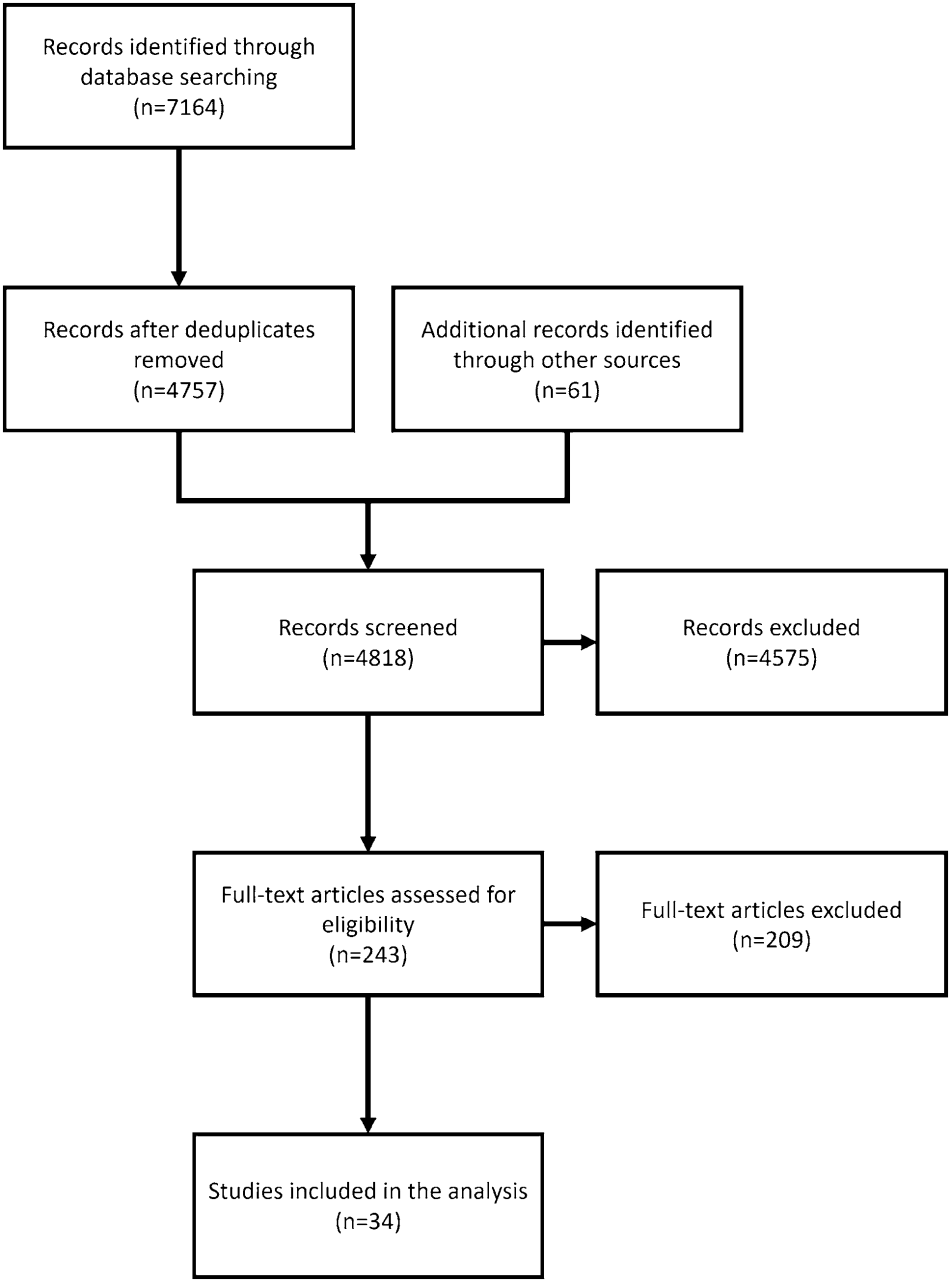
Flow diagram

## Materials and methods

A protocol for this scoping review was developed and published on the Open Science Framework, where the study was registered before performing the search strategy (https://doi.org/10.17605/OSF.IO/W2F8N) [23]. We have followed the PRISMA-ScR Statement for reporting scoping reviews [24].

### Eligibility criteria

Based on the population, concept, and context (PCC) framework for scoping reviews [25], inclusion criteria were: (1) adult (≥ 18 years) population; (2) use of the CFS; (3) emergency medicine setting (intra-hospital or pre-hospital); and (4) original research. We did not language restrict.

Studies not reporting frailty or reporting frailty using another tool (such as Fried [26], ISAR [27]) exclusively were excluded. We also excluded conference abstracts, editorials, commentaries, position papers, narrative and systematic reviews, and case studies, that did not report on original research.

### Search strategy

The MEDLINE search strategy was developed by a health science librarian and peer-reviewed by another librarian [28]. Databases searched were MEDLINE(R) ALL via Ovid, Embase Classic + Embase via Ovid, EBM Reviews—Cochrane Central Register of Control Trials via Ovid, CINAHL via EBSCOhost, Ageline via EBSCOhost, and Scopus. The main search concepts were comprised of terms related to emergency department or pre-hospital settings and frailty. The date of publication was limited from 2005 to 2021. This limit was applied as the Clinical Frailty Scale (CFS) was introduced in 2005. The search strategy was developed in MEDLINE (Appendix 1) and translated to other databases. All databases were searched on July 6th, 2021. Additionally, a manual search of all eligible articles’ reference lists was completed to identify any additional literature.

### Selection of source of evidence

Search results were imported into Covidence and de-duplicated [29]. Screening and data abstraction were also completed in Covidence. First, team members screened a sample of 50 citations. Conflicts were reviewed and discussed. As the agreement on the pilot test was low (< 90%), another pilot was performed, with success. Then, two reviewers independently screened all remaining citations. Disagreements were resolved by consensus. Second-level screening was performed using a similar strategy (pilot, double independent screening). The study screening form can be found in Appendix 2.

### Data charting process and data items

Data were abstracted, using a pre-specified data abstraction form. To ensure consistency between reviewers, all reviewers initially abstracted the same five citations. Any discrepancies were resolved by consensus. The form was then adapted (Appendix 3), and data abstraction was completed independently by two reviewers. We collected data on publication characteristics (authors, country, year of publication, journal), study characteristics (design, sample size, setting, patients’ age and sex), frailty [version of CFS used, cut-off used to define frail people, type of categorization of CFS, purpose of the assessment (outcome, screening, descriptive, exposure, covariate, potential predictor), assessor, prevalence of frailty] and outcomes under study. When composite outcomes were studied, we collected each outcome of the composite outcome individually.

### Critical appraisal of individual sources of evidence

As the main goal of this study was to report on the contextual features of frailty in emergency medicine literature, no critical appraisal was performed on the individual studies.

### Synthesis of results

Results of the search and the screening process are presented using a flow diagram. Outcomes were grouped according to essential themes for the purpose of analysis.

## Results

Figure 2 presents the study flow diagram. From the 7164 records, we identified 4757 unique citations after deduplication. Sixty-one studies were also identified from references of included articles. Following first-level screening, 4575 were deemed irrelevant. Second-level screening excluded a further 209 citations. Thirty-four manuscripts (33 full manuscript and one research letter) underwent complete data abstraction and are presented in this manuscript (Appendix 4). No potentially relevant studies were excluded.

Table 1 presents characteristics of the included studies. All studies were published in English and the primary author affiliation was mainly from North America [7, 30–43] (44%) and Europe [44–55] (35%). No papers had been published before 2015, and most of the papers (76%) were published beginning 2019. Studies were published in emergency medicine journals (41%) [7, 30, 32, 33, 36, 40, 46, 48–50, 53, 54, 56, 57], geriatric journals (38%) [31, 34, 35, 38, 39, 41, 42, 45, 47, 56, 58, 59] or other types of journals (21%) [37, 43, 51, 52, 55, 60, 61].

**Table 1:** Summary of study characteristics, *N* = 34

Study Characteristics	
Main author affiliation-*n* (%)	
North America	15 (44)
Europe	12 (35)
Oceania	4 (12)
Asia	3 (9)
Year of publication-*n* (%)	
Before 2018	6 (18)
2018	2 (6)
2019	7 (21)
2020	8 (24)
2021	11 (32)
Journal category-*n* (%)	
Emergency medicine	14 (41)
Geriatric medicine	13 (38)
Other	7 (21)
Study design-*n* (%)	
Prospective cohort	22 (65)
Retrospective cohort	8 (24)
Intervention study	3 (9)
Cross-sectional study	1 (3)
Required participant consent-*n* (%)	
No	4 (12)
Yes	20 (59)
Not reported	10 (29)
Study sample size – median (IQR)	612 (330–1309)
Female proportion – median (IQR)	55 (51–63)
Mean or median age – median (IQR)	79 (77–82)
CFS version-*n* (%)	
7 levels	6 (18)
9 levels	25 (74)
Not reported	3 (9)
Cut-off to define frailty-*n* (%)	
≥ 4	5 (14)
≥ 5	12 (35)
Not reported	15 (44)
Not applicable	2 (6)
Frailty prevalence–median (IQR)	36.8 (31.8–57.6)
Assessment purpose-*n* (%)	
Main exposure	15 (44)
Predictor	5 (15)
Outcome (including reliability studies)	5 (15)
Descriptive	3 (9)
Inclusion criteria	2 (6)
Covariate	1 (3)
Other	3 (9)

Two-thirds of the studies were prospective cohorts [7, 30, 33, 35–38, 40, 42, 43, 47–50, 52, 54–59, 62], while the remaining were retrospective cohorts (24%) [34, 39, 41, 45, 46, 53, 60, 61], intervention studies (9%) [31, 44, 51] or cross-sectional studies (3%) [32] (Table 1). One study [45] was performed in pre-hospital setting only, and another one [43] included both pre-hospital and ED patients. Overall, the median sample size was 612, with an important variability from one study to the other (IQR 330–1309). The median or mean age varied between 75 and 85, while the proportion of female patients varied between 36 and 77%. Patient consent was required in 20 studies and not required in four studies [36, 45, 53, 54]. The 10 remaining studies [32, 34, 41, 43, 46, 50, 51, 59–61] did not mention patient consent.

The majority (74%) of the studies used the nine-point CFS [32–34, 36, 39–41, 44–46, 48–62]. For three studies [30, 43, 47], it was not possible to assess which CFS version was used. Only two studies excluded patients with CFS score of nine. [33, 49] Thirteen studies reported frailty prevalence, with a median (using authors’ cut-off) of 36.8% (IQR 31.8–57.6). Frailty was assessed mostly during patient work-up (65%) [31–33, 35–38, 40, 42, 44, 45, 47–49, 51, 53, 55, 56, 58, 59, 61, 62], while some authors assessed it at triage (18%) [41, 46, 50, 52, 54, 60], at patient disposition (9%) [7, 34, 57] or at other times (9%) [30, 39, 43]. Table 2 shows the different types of assessors. Research staff (35%) [7, 30, 31, 35, 37, 38, 43, 47, 49, 56, 59, 62], nurse (32%) [36, 40, 41, 44, 46, 48, 50, 52, 54, 58, 60] and ED physician (20%) [32, 33, 36, 40, 42, 46, 57] were the most frequent.

**Table 2:** Person completing Clinical Frailty Scale assessment

Assessor	Number of studies (frequency)*
Research staff	12 (35)
Nurse	11 (32)
ED physician	7 (20)
Patients	3 (9)
Geriatric physician	2 (6)
Other	3 (9)
Not reported or unclear	3 (9)

*Total of studies can exceed number of studies as some studies used more than one type of assessor

CFS was most commonly used as a main exposure (44%) [7, 33, 37–39, 41, 42, 46, 49, 52, 53, 55, 57, 60]. Other frequent purposes included potential predictor (15%) [30, 35, 45, 56, 62] and outcome (15%) [32, 36, 40, 43, 48]. Only two studies used it as an eligibility criterion. When CFS was used as a main exposure or a predictor (20 studies), the most frequent studied outcomes (either alone or in composite) were mortality (10 studies, 50%) [33, 39, 46, 49, 55–58, 60, 62] and hospital admission (7 studies, 35%) (Table 3) [33, 35, 41, 49, 53, 55, 60]. For mortality, several time points were used, including 1 month [33, 39, 49, 55, 57, 60], 3 months [56, 62] or 1 year. Three papers used it as a time-to-event variable [39, 46, 49]. Four papers considered patient-oriented outcomes (alone or included in a composite outcome), such as quality of life [37, 58], functional decline [38, 42] or need for community service following discharge [58]. In the case of use as the main exposure, a sample size calculation was reported only in three studies [7, 49, 52]. Different methods to deal with the CFS variable as exposure or predictor were used for the statistical analysis: binarization (35%) [7, 33, 38, 55, 56, 58, 62], categorisation in 3 or more groups (30%) [35, 37, 39, 42, 46, 49] or continuous (20%) [41, 45, 53, 57]. One study [60] used different methods and two studies [30, 52] did not mention their analytic approach. Among the 15 studies looking for an association between a main exposure and an outcome, only 3 (20%) mentioned a sample size calculation [7, 49, 52]. Finally, these 15 studies found a statistically significant association. Three studies did not incorporate any covariate in the model [41, 42, 52]. For the other ones, age (10 studies [7, 33, 38, 46, 49, 53, 55, 57, 58, 60]), sex or gender (9 studies [33, 38, 46, 49, 53, 55, 57, 58, 60]) and comorbidities (7 studies [37, 38, 46, 53, 57, 58, 60]) were the most frequent covariates used for adjustment (Table 4).

**Table 3:** Reported study outcome measures

Outcomes	Number of studies (frequency)*
Mortality	10 (50)
Admission	7 (35)
Readmission or return to the ED	4 (20)
Length of stay	3 (14)
Delirium	2 (10)
Functional decline	2 (10)
ICU admission	2 (10)
Quality of life	2 (10)
Others	7 (35)

*Total of studies can exceed number of studies as some looked at more than one outcome

*N* = 20

**Table 4:** Adjusting variables,

Variables	Number of studies (frequency)*
Age	10 (67)
Sex/gender	9 (60)
Comorbidities	7 (47)
Severity/Acuity	6 (40)
At least one other	6 (40)
None	3 (20)

*Total of studies can exceed number of studies as some studies included more than one covariate

*N* = 15

## Discussion

We conducted a scoping review that explored the use of the CFS in adult patients in emergency medicine. We found there is increasing use of the CFS in the emergency setting. Most of the studies using it have been published in recent years. The revised version of the CFS with nine points was the most frequently used; however, the purpose and timing of the CFS, who performed the assessment and the analytic approach differed between studies. The cut-off used to define frailty not reported in almost half of studies and the most frequent use of CFS was as an exposure, to look at an association with an outcome.

Our study adds to the work of Church et al., and van Dam et al. [18, 63]. Van Dam et al. completed a narrative review of frailty assessment in the ED. Their study evaluated multiple tools and only included three studies that used the CFS. Church, on the other hand, focused exclusively on use of the CFS, but only six were in the ED. While there are some similarities, including trend over time, assessors and outcomes under study, our findings contribute significantly to our understanding of the current use of the CFS in the ED, as we focused on the ED setting and we examined additional characteristics, such as consent and statistical analysis.

This research showed that consent was required for study inclusion most of the time. While we acknowledge the importance to seek patient consent to participate in a study, studies looking at the impact of frailty assessment or association with outcomes that exclude patients that cannot give informed consent are at risk of, in the very least, limiting the generalizability of the results but in the worst case biasing their results. The impact of patient selection based on consent on study results has been shown in other vulnerable populations, including patients with delirium and stroke [64, 65]. As there appears to be a relation between frailty and ability to give informed consent, the risk of bias in this patient population is high [66]. Therefore, it would be optimal to get a waiver of consent for minimum risk studies.

Another important finding of this study is suboptimal reporting regarding CFS. It was occasionally difficult to determine who completed the CFS assessment, when the assessment took place, which version of the CFS was used or how the CFS was considered in the analysis. A lack of standardized reporting is a crucial issue in research as it could impact interpretation and reproducibility of results [20].

Regarding the analysis, our study highlights several issues that should be mentioned. Studies that reported frailty prevalence or used frailty as a binary variable in their analysis, did not use a consistent CFS cut-off, some authors used four and more whereas other authors used five and more, likely because of the recent change of wording (“vulnerable” to “very mild frailty”). Although binarization is never the best solution, there needs to be consensus regarding a standardized cut-off if the CFS is to be dichotomized. While many studies consider frailty as a binary variable, some authors used it as a continuous one. Such analysis should be performed with caution as it is unlikely that regression fundamental assumptions would be met, such as linearity of the log-odds. Using categories, or even more advanced methods such as restricted cubic spline, could improve the rigor in this part of a study [67]. Almost all authors chose to adjust the main association. Age and comorbidities were frequently chosen. It can be argued that, because the CFS is a multi-faceted tool, incorporating already such aspects, there is a risk of collinearity.

Some limitations of this scoping review should be acknowledged. Our search strategy was developed for our specific question, however there is the possibility that studies could have been missed, especially studies with CFS used as inclusion criteria, baseline characteristics or covariates as they are frequently not mentioned in the abstract. Therefore, the results regarding the purpose of the CFS assessment in the ED could be biased, with a risk of underestimating the use of CFS for those purposes. We decided a priori to include only studies with patients, as our goal was to see how the CFS was used in the ED. There are, however, some papers on the reliability of the CFS that were based on clinical vignettes. Those studies were excluded. Finally, to ensure the homogeneity of our results, we excluded papers that included both ED patients and ward patients, as the finding could have biased our results, if the CFS was not assessed in the ED environment.

This scoping review has strengths. To our knowledge, this is the first exhaustive review on the CFS in the ED. The results from this review will help to define future research questions. Secondly, we used rigorous methodology for the sources (several databases, published papers and conferences abstract), the search strategy (more comprehensive than previous studies), the screening (pilot testing, double independently review) and the data extraction. This process reinforces the internal validity of our results. Finally, this scoping review was registered, its protocol is available, and all amendments to this protocol are listed to increase the transparency of our work.

Based on this review, we identify several gaps that could be considered in future research projects. From a global perspective, there needs to be a move toward common data elements (including cut-off point where appropriate) and core outcome measures [68]. Consensus on data elements and outcome measures for the CFS in the ED could be achieved using the Delphi methodology [69]. We identified multiples studies that looked at the association between CFS level and outcomes. Robust synthesis, including bias assessment and meta-analysis should be performed. From a clinical perspective, there are currently few studies looking at the added value of the systematic use of the CFS in the ED. Evaluation of the impact of ED frailty screening with this tool is therefore needed. Studies comparing frailty screening to no screening are required before advocating for a large implementation of frailty screening. Other important questions include who should complete the frailty evaluation and what is the optimal timing of frailty assessment during the ED course. While it has been shown in the ICU that assessment based on chart review, with family or directly to the patient were quite similar [70], the research on this issue within emergency medicine is scarce. It is likely that assessing frailty at triage versus at disposition could have a different impact. Finally, we found only one study performed exclusively in the pre-hospital setting. When paramedic attend at patients’ home, they could have a better perspective of their environment and could therefore have a more accurate assessment of their frailty.

In summary, this scoping review found increasing use of the Clinical Frailty Scale in studies with adults presenting to the ED. The majority of studies used it as a predictor for adverse outcomes, most commonly admission to hospital and mortality. The quality of the reporting in future studies must be improved. Future research should look at how patients can benefit from its use in the ED and when, how and by whom the CFS should be used.

## Data Availability

The data that support the findings of this study are available on the Open Science Framework (https://doi.org/10.17605/OSF.IO/WQRFV).

## Appendix 1: Search strategy draft Ovid MEDLINE(R) ALL < 1946 to July 02, 2021 >

**Table:**

#	Searches	Results
1	((emergenc* or accident) adj3 (department? or room? or ward? or unit? or service? or hospital? or care? or medicine? or treatment? or technician* or practioner* or rescu* or triag*)).ti,ab,kf	180,881
2	(Out of hospital or Prehospital or pre-hospital or paramedic* or ambulance* or dispatch* or first responder*).ti,ab,kf	45,798
3	(Emergenc* adj2 (medical or health) adj2 service*).ti,ab,kf	11,447
4	"observation unit?".ti, ab, kf	886
5	exp Emergency Medical Services/	150,742
6	Emergencies/	41,625
7	exp Emergency Service, Hospital/	85,732
8	exp Emergency Medicine/	14,435
9	Emergency Medical Technicians/	5820
10	exp Emergency Treatment/	125,715
11	or/1–10	409,059
12	CFS.ti, ab, kf	7384
13	frail*.ti, ab, kf	26,761
14	Frailty/	4442
15	Frail Elderly/	12,681
16	or/12–15	38,245
17	11 and 16	1375
18	limit 17 to year = "2005-Current"	1218

## Appendix 2: Screening form

**Table:**

Question	Answer	Decision
1st-level screening (Title and abstract)		
Does the study concern emergency medicine patients (Emergency department, pre-hospital field, paramedics)?	No	Exclusion
	Yes/Unsure	Go-on screening
Does this study report original research?	No	Exclusion
	Yes/Unsure	Go-on screening
Does the title or the abstract mention CFS or frailty?	No	Exclusion
	Yes	Inclusion
2nd-level screening (Full text screening)		
Does the study report original research?	No (systematic or scoping review)	Exclusion
	No (editorial, letter, etc.)	Exclusion
	Yes (intervention, cohort, case control, secondary analysis, etc.)	Go on screening
Does the study report the assessment of frailty using the CFS (inclusion criteria, Table 1, exposure, results, etc.)?	No	Exclusion
	Yes / Doubt	Go on screening
Are the patients assessed in the pre-hospital field or in the ED?	No	Exclusion
	Doubt/Yes	Go on screening

## Appendix 3: Extraction form

**Table:**

Type	Full text / Letter
First author name	Free text
Country of first affiliation	Free text
Email of corresponding authors	Free text
Year of publication	XXXX
Journal	Free text
Study design	Not mentioned/Unclear/Intervention/Prospective cohort/retrospective cohort/Case control/Other (Free text)
Sample size	XXX
Setting	ED only/Prehospital only/Mixed/Other (Free text)
Patient’s age (mean or median)	Not mentioned/XXX
Female proportion (%)	Not mentioned/XXX
Version of CFS used	7/9/Not mentioned
Cut-off to define frail patients	Not mentioned/Free text
Purpose of the assessment	Eligibility criteria/Main exposure/Co-variate/Outcome/Predictor/Descriptive only/Other (Free text)
If main exposure or covariate, how was the variable analyzed	Continuous
	Binarization
	Categorization
	Transformed
	Other
If main exposure, sample size calculation performed	Yes/No/Not mentioned
Assessor	Not mentioned/Nurse/ED physician/Geriatric physician/Research staff/Administrative staff/Other (Free text)
Time of assessment	Triage
	Patient’s work-up
	Disposition
	Other (Free text)
Prevalence of frailty (%)	Not mentioned/XXX
Primary outcome	Not mentioned/Free text
Statistically significant association between frailty and the outcome	Not mentioned/Yes/No
Secondary outcomes	Free text
Confounders adjusted association	Yes/No
If confounders:	Free text

## Appendix 4: Studies included in the analysis

- Alakare J, Kemp K, Strandberg T, et al. Systematic geriatric assessment for older patients with frailty in the emergency department: a randomised controlled trial. *BMC Geriatrics.* 2021;21(1):1–11.
- Beland E, Nadeau A, Carmichael P–H, et al. Predictors of delirium in older patients at the emergency department: a prospective multicentre derivation study. *CJEM.* 2021;23(3):330–336.
- Bernard P, Corcoran G, Kenna L, et al. Is Pathfinder a safe alternative to the emergency department for older patients? An observational analysis. *Age and ageing.* 2021(0,375,655, 2xr).
- Boucher V, Lamontagne M-E, Lee J, Carmichael P–H, Dery J, Emond M. Acceptability of older patients' self-assessment in the Emergency Department (ACCEPTED)-a randomised cross-over pilot trial. *Age and ageing.* 2019;48(6):875–880.
- Cardona M, Lewis ET, Kristensen MR, et al. Predictive validity of the CriSTAL tool for short-term mortality in older people presenting at Emergency Departments: a prospective study. *European geriatric medicine.* 2018;9(6):891–901.
- Cardona M, O'Sullivan M, Lewis ET, et al. Prospective Validation of a Checklist to Predict Short-term Death in Older Patients After Emergency Department Admission in Australia and Ireland. *Academic emergency medicine: official journal of the Society for Academic Emergency Medicine.* 2019;26(6):610–620.
- Clark S, Shaw C, Padayachee A, Howard S, Hay K, Frakking TT. Frailty and hospital outcomes within a low socioeconomic population. *QJM: monthly journal of the Association of Physicians.* 2019;112(12):907–913.
- Dresden SM, Platts-Mills TF, Kandasamy D, Walden L, Betz ME. Patient Versus Physician Perceptions of Frailty: A Comparison of Clinical Frailty Scale Scores of Older Adults in the Emergency Department. *Academic emergency medicine: official journal of the Society for Academic Emergency Medicine.* 2019;26(9):1089–1092.
- Elliott A, Taub N, Banerjee J, et al. Does the Clinical Frailty Scale at Triage Predict Outcomes From Emergency Care for Older People? *Annals of emergency medicine.* 2021;77(6):620–627.
- Fernando SM, Guo KH, Lukasik M, et al. Frailty and associated prognosis among older emergency department patients with suspected infection: A prospective, observational cohort study. *CJEM.* 2020;22(5):687–691.
- Giroux M, Sirois M-J, Boucher V, et al. Frailty Assessment to Help Predict Patients at Risk of Delirium When Consulting the Emergency Department. *The Journal of emergency medicine.* 2018;55(2):157–164.
- Griffin A, O'Neill A, O'Connor M, Ryan D, Tierney A, Galvin R. The prevalence of malnutrition and impact on patient outcomes among older adults presenting at an Irish emergency department: a secondary analysis of the OPTI-MEND trial. *BMC geriatrics.* 2020;20(1):455.
- Hominick K, McLeod V, Rockwood K. Characteristics of Older Adults Admitted to Hospital versus Those Discharged Home, in Emergency Department Patients Referred to Internal Medicine. *Canadian geriatrics journal: CGJ.* 2016;19(1):9–14.
- Ishikawa S, Miyagawa I, Kusanaga M, et al. Association of frailty on treatment outcomes among patients with suspected infection treated at emergency departments. *European journal of emergency medicine: official journal of the European Society for Emergency Medicine.* 2021;28(4):285–291.
- Jarman H, Crouch R, Baxter M, et al. Feasibility and accuracy of ED frailty identification in older trauma patients: a prospective multi-centre study. *Scandinavian journal of trauma, resuscitation and emergency medicine.* 2021;29(1):54.
- Kaeppeli T, Rueegg M, Dreher-Hummel T, et al. Validation of the Clinical Frailty Scale for Prediction of Thirty-Day Mortality in the Emergency Department. *Annals of emergency medicine.* 2020;76(3):291–300.
- Kemp K, Alakare J, Harjola V-P, et al. National Early Warning Score 2 (NEWS2) and 3-level triage scale as risk predictors in frail older adults in the emergency department. *BMC emergency medicine.* 2020;20(1):83.
- Lee J, Sirois M-J, Moore L, et al. Return to the ED and hospitalisation following minor injuries among older persons treated in the emergency department: predictors among independent seniors within 6 months. *Age and ageing.* 2015;44(4):624–629.
- Lewis ET, Dent E, Alkhouri H, et al. Which frailty scale for patients admitted via Emergency Department? A cohort study. *Archives of gerontology and geriatrics.* 2019;80(8,214,379, 7ax):104–114.
- Liu H, Shang N, Chhetri JK, et al. A Frailty Screening Questionnaire (FSQ) to Rapidly Predict Negative Health Outcomes of Older Adults in Emergency Care Settings. *The journal of nutrition, health & aging.* 2020;24(6):627–633.
- Lo AX, Heinemann AW, Gray E, et al. Inter-rater Reliability of Clinical Frailty Scores for Older Patients in the Emergency Department. *Academic emergency medicine: official journal of the Society for Academic Emergency Medicine.* 2021;28(1):110–113.
- McGrath J, Almeida P, Law R. The Whittington Frailty Pathway: improving access to comprehensive geriatric assessment: an interdisciplinary quality improvement project. *BMJ open quality.* 2019;8(4):e000798.
- O'Caoimh R, Costello M, Small C, et al. Comparison of Frailty Screening Instruments in the Emergency Department. *International journal of environmental research and public health.* 2019;16(19).
- O'Shaughnessy I, Romero-Ortuno R, Edge L, et al. Home FIRsT: interdisciplinary geriatric assessment and disposition outcomes in the Emergency Department. *European journal of internal medicine.* 2021;85(9,003,220):50–55.
- Provencher V, Sirois M-J, Emond M, et al. Frail older adults with minor fractures show lower health-related quality of life (SF-12) scores up to six months following emergency department discharge. *Health and quality of life outcomes.* 2016;14(101,153,626):40.
- Provencher V, Sirois M-J, Ouellet M-C, et al. Decline in activities of daily living after a visit to a Canadian emergency department for minor injuries in independent older adults: are frail older adults with cognitive impairment at greater risk? *Journal of the American Geriatrics Society.* 2015;63(5):860–868.
- Pulok MH, Theou O, van der Valk AM, Rockwood K. The role of illness acuity on the association between frailty and mortality in emergency department patients referred to internal medicine. *Age and ageing.* 2020;49(6):1071–1079.
- Ringer T, Thompson C, McLeod S, Melady D. Inter-rater Agreement Between Self-rated and Staff-rated Clinical Frailty Scale Scores in Older Emergency Department Patients: A Prospective Observational Study. *Academic emergency medicine: official journal of the Society for Academic Emergency Medicine.* 2020;27(5):419–422.
- Serina P, Lo AX, Kocherginsky M, et al. The Clinical Frailty Scale and Health Services Use for Older Adults in the Emergency Department. *Journal of the American Geriatrics Society.* 2021;69(3):837–839.
- Shen VW-C, Yang C, Lai L-L, et al. Emergency Department Referral for Hospice and Palliative Care Differs among Patients with Different End-of-Life Trajectories: A Retrospective Cohort Study. *International journal of environmental research and public health.* 2021;18(12).
- Shrier W, Dewar C, Parrella P, Hunt D, Hodgson LE. Agreement and predictive value of the Rockwood Clinical Frailty Scale at emergency department triage. *Emergency medicine journal: EMJ.* 2020(b0u, 100,963,089).
- Simon NR, Jauslin AS, Rueegg M, et al. Association of Frailty with Adverse Outcomes in Patients with Suspected COVID-19 Infection. *Journal of clinical medicine.* 2021;10(11).
- Sirois M-J, Griffith L, Perry J, et al. Measuring Frailty Can Help Emergency Departments Identify Independent Seniors at Risk of Functional Decline After Minor Injuries. *The journals of gerontology Series A, Biological sciences and medical sciences.* 2017;72(1):68–74.
- Tong T, Chignell M, Tierney MC, et al. Technology profiles as proxies for measuring functional and frailty status. *Procedia Comput Sci.* 2017;111(C):77–86.

## Declarations

### Previous presentation

The manuscript was previously presentend in SAEM2022, New-Orleans, Louisiane, USA, May 2022.

### Amendments

Some amendments were done to our protocol. We dropped the language restriction and we adapted screening form and data extraction form following initial training.
